# Maternal mortality at muhimbili national hospital in Dar-es-Salaam, Tanzania in the year 2011

**DOI:** 10.1186/1471-2393-14-320

**Published:** 2014-09-13

**Authors:** Andrea B Pembe, Chetto Paulo, Brenda S D’mello, Jos van Roosmalen

**Affiliations:** Department of Obstetrics and Gynaecology, School of Medicine, Muhimbili University of Health and Allied Sciences, Dar es Salaam, Tanzania; Dodoma Regional Referral Hospital, Dodoma, Tanzania; Maternal and Newborn Health Care, Comprehensive Community Based Rehabilitation in Tanzania, Dar es Salaam, Tanzania; Department of Obstetrics, Leiden University Medical Centre, Leiden, The Netherlands; Department of Medical Humanities, EMGO Institute for Health and Care Research, VU University Medical Centre Amsterdam, Amsterdam, The Netherlands

## Abstract

**Background:**

Improving maternal health is one of the eight millennium development goals adopted at the millennium summit in the year 2000. Within this frame work, the international community is committed to reduce the maternal mortality ratio by 75% between 1990 and 2015. The objective of this study was to determine the maternal mortality ratio, classify causes of maternal deaths and assess substandard care factors at Muhimbili National Hospital (MNH), Dar-es-Salaam in Tanzania.

**Methods:**

A retrospective review of all maternal death records of cases that occurred from 1^st^ January to 31^st^ December 2011 was done.

**Results:**

There were 10,057 live births, 155 maternal deaths and hence MMR of 1,541 per 100,000 live births. Direct causes of maternal deaths were classified in 69.5% of the maternal deaths. Of the direct causes, preeclampsia/eclampsia was the major cause (19.9% of all deaths), followed by post partum haemorrhage (14.9%), abortion complications (9.9%) and sepsis (9.2%). Among the indirect causes anaemia was the leading cause (11.3%) of all deaths, followed by HIV/AIDS (9.9%). Substandard care factors contributing to deaths were identified in 116 (82.3%) of all cases. Among these 28 had patient factors only, 71 medical service factors while 17 had both patient and medical service substandard care factors. The common factors from the woman’s side included delay in seeking care (73.3%) and complete lack of antenatal care (11.1%). Of the medical service factors, inadequate (26.1%) or no blood for transfusion (19.3%), delay in receiving treatment (18.3%) and mismanagement (17%) were the common factors.

**Conclusion:**

There is a high maternal mortality ratio at MNH. Hypertensive disorders of pregnancy, post partum haemorrhage and anaemia are the leading causes of maternal deaths in this institution. Multiple substandard care factors identified both at individual and health care service levels that contributed to maternal deaths. There is a need for increasing efforts in the fight to reduce maternal deaths at the institution. A more pro-active role from the referring facilities in the region is needed.

## Background

Globally, 287,000 women die because of the complications related to pregnancy and childbirth [1]. Sub-Saharan Africa with only 20% of the world’s child birth, accounts for about half of these deaths [2]. The life time risk of maternal death is 1 in 31 in Africa compared to 1 in 4300 in high income countries [1]. Tanzania is among the eleven countries in the world which comprised 65 percent of all maternal deaths in 2008 [1]. According to Tanzania Demographic and Health Surveys of 2005 and 2010, maternal mortality ratio has declined from 578 per 100,000 in 2005 [3] to 454 per 100,000 live births in 2010 [4]. This is an improvement, but the figure is still high and far beyond the fifth millennium development goal (MDG 5) target of reducing maternal mortality ratio to 193 per 100,000 live births by 2015.

Although indirect causes of maternal mortality are on the increase in Tanzania, direct causes are still the majority [5]. The commonest are haemorrhage, sepsis, hypertensive disorders and eclampsia, abortion complications and obstructed labour. Thaddeus and Maine developed a three phases delay model to explain factors which affect access to effective interventions to prevent maternal and neonatal deaths when complications occur [6]. The first is delay in making the decision to seek care because of failure to recognize complications. The second is delay in reaching care, due to poor roads and geographical barriers. The third is delay in receiving care in health facilities. The third delay can be reduced if all women with complications related to pregnancy arriving at health facilities receive quality emergency obstetric and neonatal care.

Most maternal deaths in Tanzania occur after the mothers have had contact with health care facilities [7]. This means that these mothers were more likely to survive if timely adequate interventions could have been provided in the facilities. Muhimbili National Hospital (MNH) has a maternal death review committee which is responsible for reviewing all cases of maternal deaths. The committee has six members comprising of four specialists, one of whom is an anaesthesiologist and two are senior nurse midwives. The committee is supposed to meet once every week and review maternal deaths using a structured review form. It was observed that the committee did not meet regularly as planned for the year 2011. It reviewed only fifteen maternal deaths [8]. This prompted us to design this study with the objective to determine the maternal mortality ratio, classify the causes and assess substandard care factors of maternal deaths at MNH. This will be useful for planning interventions to reduce maternal morbidity and mortality in the institution and its referring facilities in the region.

## Methods

### Study design

A retrospective case review of all maternal deaths occurred from 1^st^ January to 31^st^ December 2011 was done.

### Study setting

Muhimbili National Hospital is the largest referral hospital in Tanzania and a teaching hospital for Muhimbili University of Health and Allied Sciences. It is located in Dar-es-Salaam city with a population of 4.5 million. Dar-es-Salaam city is divided into three Municipals, each with one public hospital which provides obstetrical and gynaecological services. The hospital receives maternal cases mostly from the Municipal hospitals and the districts of the Coast region. Occasionally patients are received from upcountry.

Women with pregnancies or having delivered pregnancies of 28 weeks or more are admitted in the maternity block. Maternity block has seven wards. Four wards are reserved for admission of women with antenatal and postnatal complications and women with sick children. Additionally there is a ward for neonatal admissions, a labour ward and a postnatal ward for women with uneventful spontaneous vaginal deliveries with normal babies. This postnatal ward has an area reserved as intensive care-like unit for patients with severe preeclampsia and eclampsia. The labour ward has 20 delivery beds and a total of 25 nurse midwives. Everyday there are three shifts of nurse midwives each with an average of five. Admitted women are attended by a team of doctors on duty including intern, registrar or resident doctors, and obstetrics and gynaecology specialists. The hospital has a theatre with two operating rooms adjacent to labour ward where about 8 to 12 operative deliveries both emergences and elective are performed each day. The number of deliveries per day is between 20 and 40.

There are two gynaecological wards, each with 32 beds. The gynaecological wards admit women with pregnancies of less than 28 weeks of gestation who have developed complications together with non pregnant women with gynaecological conditions. Emergency operations are conducted either in the Emergency medicine operation theatre or a gynaecological operating room at the main theatre.

### Data collection

The record books of the gynecological, obstetrical and labour wards, and operating theatres were checked for cases of maternal deaths to ensure that all deaths are identified. The hospital’s obstetrical electronic data base was as well included. The names and registration numbers were identified and these were used to retrieve the patients’ case files in the medical records department.

Maternal death was defined according to the International Classification of Diseases and related health conditions 10^th^ revision (ICD 10), in which maternal death is defined as death of a woman while pregnant or within 42 days of the termination of pregnancy irrespective of the duration and the site of pregnancy, from any cause related to or aggravated by the pregnancy or its management but not from accidental or incidental causes. This definition allows identification of maternal deaths, based on their causes as either direct or indirect. A direct obstetric death is defined as that resulting from obstetric complications during pregnancy, labour and puerperium, from interventions, omissions, incorrect treatment, or from a chain of events resulting from any of the above. Indirect obstetric deaths are those resulting from previous existing disease or disease that developed during pregnancy and which was not due to direct obstetric causes, but was aggravated by physiologic effects of pregnancy.

A checklist was used to collect information from the case files. The information collected included age, education status, parity, number of live births, marital status, booking status, referral status, referral diagnosis, length of hospital stay, mode of delivery, interventions in the hospital before death and the condition of the women on admission (critically ill and not critically ill). Critically ill women were those admitted with imminent risk of death including gasping, unconsciousness and deteriorated vital signs. The underlying cause of death was determined and in case of two or more possible causes of deaths, priority was given to the primary cause, judged on the basis of available clinical information, investigations and diagnosis.

Two external reviewers, the third and fourth authors, both being senior obstetricians, reviewed the case files to identify substandard care factors and the cause of maternal death. The final decision of substandard care factors and cause of death was reached after comparing it with review done by the first and second authors. In cases of incongruence a discussion was conducted and an agreement reached. Guided by the World Health Organization recommendation for the audit of the maternal deaths [9] criteria for identifying substandard factors was created. A substandard care factor was defined as deficiencies in the medical care process that may have contributed to maternal death including the patient factors. In much of the literature patient factors are also considered to be substandard care.

Substandard care factors were divided into patient factors and medical service factors. Patient factors included home delivery while having previous bad obstetric history/event, complete lack of antenatal care and insufficient antenatal care visits. Insufficient antenatal care was defined as focused antenatal care of less than 4 visits for women with no risk or complication and those women with complications who did not attend according to schedule recommended by health providers. Other patient factors included poor compliance with treatment such as refusal to take medications or refusal to undergo admission or surgery. Moreover delay in seeking care such as admission in the hospital while in critical condition like shock, coma, gasping stage or HIV/AIDS stage four was considered to have experienced a delay in seeking care. Medical service factors included inadequate blood transfusion or complete lack of blood, delay in receiving treatment when a correct diagnosis has been made, and lack of medications when the medication were not available in the facility and the patient did not get them. Other medical service factors included ‘poor management’ when the patient was inadequately treated despite having a correct diagnosis, ‘delayed investigations’ when the investigations took longer time thus contributed to delay in further management and ‘delayed diagnosis’ when there was delay in reaching a correct diagnosis. In some of the cases, women had both patient and medical service substandard care factors identified.

### Data analysis

Data collected was entered into computer using EPI info version 3.5.1 (CDC, Atlanta, Georgia, USA) which allows double entry and validation. Data was transferred to SPSS version 15.0 (SPSS Inc., Chicago, IL, USA) for analysis. Frequency distribution and measure of location were used to summarize data.

### Ethical considerations

The study was approved by the institutional review board (Senate Research and Publication Committee) of Muhimbili University of Health and Allied Sciences, and the permission to conduct the study was given by the Executive Director of MNH. This was a retrospective review of case notes of patients died of maternal complications therefore consent from the women were not obtained. To maintain anonymity and confidentiality patients’ names and information remained highly confidential and were used by the research team only.

## Results

During the study period, 10,870 deliveries occurred with 10,057 live births and 155 maternal deaths. The maternal mortality ratio thus was 1,541 per 100,000 live births. Fourteen case files were missing, leaving 141 case files for review for substandard care factors and classification.

The median age of the deceased women was 31 years (range: 17 to 45). The age group 25–29 years constituted almost a third of them. Majority had primary education (82.3%) and were married or cohabiting (88.7%). Half of them were housewives. One third of the deceased women were primigravidas. Twenty percent of the deceased women were HIV positive while more than one quarter had an unrecorded HIV status (Table 1).

**Table 1:** **Socio-demographic and obstetrical characteristics, and HIV and referral status of the deceased women, (N = 141)**

Characteristic	Number	Percent
Age (Years)		
<20	14	9.9
20-24	33	23.4
25-29	43	30.5
30-34	29	20.6
≥ 35	22	15.6
Level of education		
No formal education	9	6.4
Primary education	116	82.3
Secondary education or higher	16	11.3
Marital status		
Single	14	9.9
Married/cohabiting	125	88.7
Divorced	2	1.4
Occupation		
Housewife	76	53.9
Petty trader	30	21.3
Peasants	28	19.9
Employee (Paid contract)	7	5
Gravidity		
1	46	32.6
2	31	22
3	27	19.1
4	20	14.2
≥ 5	17	12.1
HIV status		
HIV Positive	29	20.6
HIV Negative	72	51.1
Not known	40	28.4
Referral from^a^		
Municipal hospitals	98	77.2
Other health facilities^b^	29	22.8

^a^14 deceased mothers were attending care at Muhimbili National Hospital.

^b^This includes hospitals in Coast region and private hospitals and health centres in Dar-es-Salaam.

Seventy seven (54.6%) deceased women were critically ill on admission. Eight of the critically ill women were gasping and died on arrival. One hundred and twenty seven (90.1%) women were referred to MNH from other hospitals and health centres. Three-quarters of the referrals were from the Dar-es-Salaam Municipal hospitals while another quarter is from the district hospitals of Coast region, private hospitals and health centres of Dar-es-Salaam city. Table 2 shows reason for referral to MNH. Most of the deceased women were referred because of lack of blood, followed by lack of personnel, lack of supplies and equipments and for intensive care.

**Table 2:** **Reason for Referral to Muhimbili National Hospital, (N = 127)**

Reason for referral	Number	Percent
No blood available	38	29.9
Lack of personnel	34	26.8
Lack of supplies and equipments	33	25.9
For intensive unit care	14	11
Unrecorded	8	6.3

Seventy two (51.1%) maternal deaths occurred during the first twenty four hours of admissions, 16 (11.3%) between 25 – 48 hours, 8 (5.7%) between 49 – 72 hours and 45 (31.9%) more than 72 hours after admission.

Direct causes were classified in 69.5% of the reviewed maternal deaths while 30.5% were classified to be due to causes not directly related to pregnancy. Of the direct causes preeclampsia/eclampsia was the commonest cause of death in all deceased women, followed by post partum haemorrhage. The predominant cause of indirect maternal deaths was anaemia (11.3% of all deaths and 37.2% among indirect causes), followed by HIV/AIDS with 9.9% of all deaths and 32.6% among the indirect causes (Table 3). There were no deaths due to ectopic pregnancy in this study.

**Table 3:** **Direct and indirect causes of maternal deaths at Muhimbili National Hospital**

Characteristic	Number	Percent of all causes (N = 141)	Percent of direct/indirect cause
Direct causes			
All direct causes	98	69.5	
Preeclampsia/eclampsia	28	19.9	28.6
Postpartum haemorrhage	21	14.9	21.4
Abortion complications	14	9.9	14.3
Sepsis	13	9.2	13.3
Antepartum haemorrhage	10	7.1	10.2
Ruptured uterus	7	5	7.1
Obstructed labour	5	3.5	5.1
Indirect causes			
All indirect cause	43	30.5	
Anaemia	16	11.3	37.2
HIV related	14	9.9	32.6
Heart diseases	8	5.7	18.6
Malaria	4	2.8	9.3
Tuberculosis	1	0.7	2.3

A total of 116 (82.3%) of the deceased women had substandard care factor identified, among these 28 had a patient factor only and 71 had a medical service factor only. Seventeen cases had both patient and medical service factors (Table 4). Delay in seeking care (73.3%) was a predominant substandard care factor on the part of the patient factors, followed by complete lack of antenatal care (11.1%) and poor compliance to treatment (6.7%). Of the medical service factors commonest were complete lack of blood (19.3%) or inadequate blood transfusion (26.1%), delay in receiving appropriate treatment (18.3%) and poor management (17.0%).

**Table 4:** **Patient and medical service substandard care factors identified to contributing to maternal deaths (N = 133)**

Substandard care factor*	Number	Percent
Patient factors (n = 45)		
Delay in seeking care	33	73.3
Lack of antenatal care visit	5	11.1
Poor compliance to treatment	3	6.7
Insufficient antenatal care	2	4.4
Home delivery with previous bad outcome	2	4.4
Medical service factors (n = 88)		
Inadequate blood transfusion	23	26.1
Complete lack of blood	17	19.3
Delay in receiving appropriate treatment	16	18.3
Poor management	15	17
Delayed investigation	9	10
Delayed diagnosis	6	6.8
Lack of medications	2	2.3

*Substandard care factors occurred in 116 maternal deaths.

## Discussion

Analyses of maternal health data to assess maternal mortality, causes of maternal deaths and substandard care factors are among the gauges of the quality of health care in a particular health facility. This study provides important information about maternal mortality in Tanzania’s largest tertiary hospital during the year 2011.

In this study maternal mortality ratio was found to be 1,541 per 100,000 live births which is three times higher than the national estimate of 454 per 100,000 live births [4]. This high MMR may be inherent to hospital based MMRs due to high risk status and complicated cases of women delivering in hospital [10]. The findings are comparable to other facility based studies in Pakistan and Nigeria where MMRs were found to be 1,650 and 1,747 per 100,000 live births respectively [11, 12]. Other studies in Nigeria and Kenya, however, showed lower MMRs of 740 and 426 respectively [13, 14]. The high MMR at MNH among other reasons, may be a result of delay in referrals with complications from other health facilities. However, this may also imply that the overall quality of maternal care in this hospital, the municipal hospitals and their catchment area is poor and the present strategies to make pregnancy safer are yet to achieve the desired changes.

Ninety per cent of maternal deaths were referred to MNH. Two-thirds of these referrals were from the Dar-es-Salaam city municipal hospitals. Lack of blood, personnel, lack of supplies and equipments were found to be the main reasons for referral from these hospitals. This questions the proper function of comprehensive emergency obstetric care services in the municipal hospitals. Another possibility is the delay of women to report to the municipal hospitals when emergencies occur. Though there may be a number of other areas that could be involved, there is a need to improve the readiness of the hospitals to provide comprehensive emergency obstetric and neonatal care including review of clinical guidelines and management protocols, and at the same time updating the staff on management of complications of pregnancy. Furthermore, as there are no clear guidelines on indications for referrals from municipal hospitals to MNH, this needs to be developed and implemented to reduce unnecessary referrals and prevent undue delays which endanger women’s life.

Half of the maternal deaths occurred within the first twenty four hours of admission to MNH. These findings are similar to a study at Jos University teaching hospital in Nigeria [15]. Early deaths in the referred hospital may reflect the possibility of delay of women with complications to report to hospital (Phase one and two delays) or delay to receive appropriate treatment at the referring health facilities and at MNH as well (Phase three delays).

Direct causes of maternal deaths in our study accounted for about two third of maternal deaths while indirect causes were found in one third. The direct causes of maternal death are treatable and death can be prevented if timely and appropriate treatment is provided when the complication occurs. We found the same major five direct causes as in other studies in sub-Saharan Africa and Asia [14, 16–18].

Severe anaemia and HIV/AIDS were the leading causes of indirect maternal deaths. Similar findings have been reported in Africa [13–15]. Anaemia is highly prevalent in Tanzania, reported in up to 60% among pregnant women while 4% have severe anaemia [19]. It has been reported that anaemia is an underlying risk factor for maternal mortality [20, 21]. HIV/AIDS is associated with opportunistic infections resulting in maternal deaths [13]. Attending antenatal clinics early in pregnancy and accepting preventive measures for anaemia including de-worming and intermittent preventive treatment for malaria, and starting PMTCT plus recommended treatment when HIV-positive, may play part in reduction of maternal deaths.

Majority of the maternal deaths had substandard care factors identified. Patient factors accounted for about one third of substandard care factors, similar to other studies from sub-Saharan Africa [22, 23]. This reflects the importance of interventions which address the first two delay components: recognizing complications and making decision to seek medical attention and also delay in reaching care due to poor infrastructure.

A high number of medical service factors were identified in this study including administrative factors such as inadequate or no blood for transfusion. Even though the country’s ministry of health has established zonal blood banks which are expected to serve all hospitals in their zones, but shortage of blood both in MNH and in referring centres is still too frequent. This is a critical issue that needs to be addressed at both the policy and community levels to ensure adequate and constant supply of blood to the hospitals. A hypothethical study in the Netherlands showed an increase of MMR from 13 to 87 per 100,000 live births (RR 6.5; 95% CI 4.2-10.0), when that country would have similar blood shortages as in sub-Saharan Africa [24]. Other medical service factors include delay in receiving treatment in health facilities (poor management, delayed investigation, delayed diagnosis and lack of medication). Studies done in other parts of Tanzania and elsewhere in Africa have shown similar factors contributing to maternal deaths [11, 22, 23, 25]. Increased investment in training and retaining skilled staff, ensuring availability and supervising the implementation of updated evidenced based protocols of care and adequate equipment and supplies are urgently needed.

This study also had some limitations indwelled in its retrospective nature. First, not all case files were available and in those available some had incomplete documentation. Though we tried to obtain missing information in many ways like using record books of each ward and obstetric data base, lack of a standard way of maintaining medical records posed a challenge. Secondly, official maternal deaths review forms from the hospital were very few as the committee did not review all deaths. The findings in this study provide some opportunities for devising solutions including strengthening the maternal death review team to help supporting intervention planning and follow up, so that each identified substandard care factor can be addressed in time. This study contributes more generally to understanding some of the challenges impeding Tanzania’s progress towards achieving MDG 5.

## Conclusion

In conclusion, MNH has a high MMR. The direct causes accounted for more than two third and the most common were hypertensive disorders of pregnancy and postpartum haemorrhage. While the most common indirect causes were anaemia and HIV/AIDS. More than half of maternal deaths occurred within twenty four hours of admission. More than two third of all referral cases were from Dar-es-Salaam municipal hospitals and the main reasons for referral were for blood transfusion and for expert management. Substandard care factors, with medical service factors contributed to a larger percentage to facility deaths than did patient level factors. This calls for improving the management of life threatening complications, both in the referring centres and in MNH. Improvement in availability of adequate blood, and blood transfusion services as well ensuring availability of adequate essential equipment and supplies to receive and manage obstetrics emergencies promptly. The maternal mortality committee of the MNH has to be revived reviewing maternal deaths regularly and the review findings translated into better care protocols and systems are required to improve the quality of maternity care services.

Further research is recommended in order to understand community level factors associated with maternal mortality by doing prospective audit of all maternal deaths and near misses in Dar-es-Salaam.
